# Corpus Callosum Radiomics-Based Classification Model in Alzheimer's Disease: A Case-Control Study

**DOI:** 10.3389/fneur.2018.00618

**Published:** 2018-07-26

**Authors:** Qi Feng, Yuanjun Chen, Zhengluan Liao, Hongyang Jiang, Dewang Mao, Mei Wang, Enyan Yu, Zhongxiang Ding

**Affiliations:** ^1^Bengbu Medical College, Bengbu, China; ^2^Department of Radiology, Zhejiang Provincial People's Hospital, People's Hospital of Hangzhou Medical College, Hangzhou, China; ^3^GE Healthcare Life Sciences, Guangzhou, China; ^4^Department of Psychiatry, Zhejiang Provincial People's Hospital, People's Hospital of Hangzhou Medical College, Hangzhou, China; ^5^Department of Radiology, Affiliated Hangzhou First People's Hospital, Zhejiang University School of Medicine, Hangzhou, China

**Keywords:** magnetic resonance imaging, Alzheimer's disease, corpus callosum, radiomics, neuroimaging

## Abstract

**Background:** Alzheimer's disease (AD) is a progressive neurodegenerative disease that causes the decline of some cognitive impairments. The present study aimed to identify the corpus callosum (CC) radiomic features related to the diagnosis of AD and build and evaluate a classification model.

**Methods:** Radiomics analysis was applied to the three-dimensional T1-weighted magnetization-prepared rapid gradient echo (MPRAGE) images of 78 patients with AD and 44 healthy controls (HC). The CC, in each subject, was segmented manually and 385 features were obtained after calculation. Then, the feature selection were carried out. The logistic regression model was constructed and evaluated according to identified features. Thus, the model can be used for distinguishing the AD from HC subjects.

**Results:** Eleven features were selected from the three-dimensional T1-weighted MPRAGE images using the LASSO model, following which, the logistic regression model was constructed. The area under the receiver operating characteristic curve values (AUC), sensitivity, specificity, accuracy, precision, and positive and negative predictive values were 0.720, 0.792, 0.500, 0.684, 0.731, 0.731, and 0.583, respectively.

**Conclusion:** The results demonstrated the potential of CC texture features as a biomarker for the diagnosis of AD. This is the first study showing that the radiomics model based on machine learning was a valuable method for the diagnosis of AD.

## Introduction

Alzheimer's disease (AD) is a progressive neurodegenerative disease, resulting in the decline of some cognitive impairments that in turn can influence the immediate and delayed memory, language, calculation, attention, and visuospatial abilities. A definitive diagnosis of AD depends on the pathological findings from an invasive autopsy or biopsy that might not be available. Therefore, noninvasive and accurate AD diagnosis is critical. Although current pharmacotherapy cannot cure this disease, early intervention can delay the disease progression and also prolongs the lives of patients with AD.

The corpus callosum (CC) is the largest white matter tract in the human brain, which connects the two hemispheres that is essential for several neurological functions, including integration of lateralized sensory input, regulation of higher-order cognitive, social function, and emotional processing (1). The CC atrophy has been found in patients with AD (2), and some of these studies have indicated that the CC atrophy might be related to the degree of cognitive impairment. Therefore, the CC atrophy might be ascribed as the neuroanatomy basis for memory decline in AD. Additionally, a recent study showed the relationship between the CC and AD by texture analysis (3).

Radiomics is a newly developed tumor diagnosis and auxiliary detection technique in recent years. It transforms the visual image information into deep features for quantitative research. Radiomics may provide almost unlimited feature information. The information includes the density, shape, size, and texture of the tumor as determined by phenotype and microenvironment, which aids in the evaluation of the efficacy and prognosis in tumor therapy. The radiomics analysis has been applied to various tumor diseases, such as glioma (4), nasopharyngeal cancer (5), breast cancer (6), hepatocellular carcinoma (7), lung cancer (8), and rectal cancer (9). Nowadays, radiomics is also applied in non-tumor areas, for example, attention-deficit hyperactivity disorder (10), Meniere's disease (11), and autism spectrum disorder (12).

In recent years, the most commonly used imaging method in radiomics studies is computed tomography (CT) that quantifies the tissue density. However, as compared to CT, magnetic resonance (MR) images can provide numerous sequences. It reflects not only the structure of the organization but also the functional metabolism and dynamic changes. MR imaging provides an enhanced tissue contrast, has a multidimensional volume, and does not require a radiation dose (13). Several MR methods have been used to study AD, including resting-state functional MRI (14), voxel-based morphometry (15), diffusion tensor imaging (16), and arterial spin labeling (17) among others. Although these methods are greatly valuable in the diagnosis of AD, they are rarely used in the related radiomics features.

In the present study, we used the T1-weighted MR images of the brain for radiomics analysis. A series of characteristics are obtained by analyzing the heterogeneity of the target area. Finally, clinical prediction and feature analysis were realized. Subsequently, we focused on studying the CC as it occupies a crucial position in AD and can be considered suitable for radiomics analysis. Therefore, the CC heterogeneity is investigated to construct a classification model for distinguishing between the patients with AD and HC.

## Materials and methods

### Patient population and data acquisition

AD subjects were recruited from the Zhejiang Provincial People's Hospital from September 2016 to February 2018. The healthy control (HC) subjects were right-handed volunteers and recruited from the health promotion center of the hospital. All the subjects provided written informed consent. This prospective study was approved by the local Ethics Committee of the hospital (No. 2012KY002). The work has been carried out in accordance with the Declaration of Helsinki.

The patients underwent a set of standard dementia screening including medical history, neuropsychological testing, physical examinations, laboratory tests, and conventional brain MRI scans. Patients with AD were first diagnosed and were required to fulfill the criteria of the revised NINCDS-ADRDA (National Institute of Neurological and Communicative Disorders and Stroke and the Alzheimer's Disease and Related Disorders Association) (18). The subjects were evaluated using the Mini-Mental State Examination (MMSE) (19). Patients with AD received an MMSE score of ≤ 24.

The criteria for HC subjects were as follows: (1) no neurological or psychiatric disorders such as stroke, epilepsy, or depression; (2) no neurological deficiencies such as hearing or visual loss; (3) no infarction, hemorrhage, or tumor lesion on conventional brain MRI; (4) achieved an MMSE score ≥ 28.

The exclusion criteria for all the subjects were as follows: (1) vascular dementia or mixed dementia; (2) stroke; (3) cerebral trauma; (4) disorders that cause memory loss such as brain tumor, epilepsy, Parkinson's disease; (5) systemic diseases such as severe anemia, diabetes, and hypertension; (6) history of administering psychoactive substances or alcohol dependence. Therefore, 85 patients with AD and 50 HC subjects were recruited initially, followed by an MRI-based examination, and those with unusable data due to the head movement were excluded (7 patients in the AD group and 6 controls). Thus, 78 patients with AD and 44 HC subjects were ultimately included in the study.

All examinations were performed using an MR scanner (Discovery MR750 3.0T; GE Healthcare, Waukesha, WI, USA). The three-dimensional T1-weighted magnetization-prepared rapid gradient echo (MPRAGE) sagittal images were collected. The scan parameters were as follows: TR = 6.7 ms, TE = 2.9 ms, TI = 450 ms, FOV = 256 × 256 mm^2^, flip angle = 12°, slice thickness/gap = 1/0 mm, in-plane resolution = 256 × 256, and 192 sagittal slices in total. All collected data is from only one MR scanner.

### Segmentation

The CC is considered the region of interest (ROI). The manual segmentation of the CC was carried out using the software “ITK-SNAP” (http://www.itksnap.org/). We selected 9 sections from each image sequence in the sagittal view: the central section, 4 to the right and 4 to the left, as the boundary of CC can be recognizable easily in the sagittal images. Consequently, the segmentation was based on anatomy, which was supported by a previous study (20). All segmentations were conducted by a radiologist and checked by an expert neuroradiologist. The differences in the opinions were resolved by integrating another expert neuroradiologist's opinion. Artificial Intelligence Kit (A.K) is a software developed by GE Healthcare Life Sciences for feature extraction and analysis. It can be combined with software “ITK-SNAP” to obtain 3D images.

### Feature calculation

First, we loaded the original three-dimensional T1-weighted MPRAGE data and ROI images in bulk into the A.K software. Then, the features including Histogram, Formfactor, Haralick, gray level co-occurrence matrix (GLCM), and gray level run-length matrix (RLM), desired for computation were selected in the data selection window. The displacement vectors were selected as 1, 4, and 7 in the relevant window. The histogram parameters were concerned with the properties of individual pixels that described the distribution of the voxel intensities in the image via basic metrics. The Formfactor parameters include descriptors of the three-dimensional shape and size of the tumor ROI. The texture is one of the major characteristics in identifying the ROI in an image. Texture represents the appearance of the surface and the distribution pattern of the voxels. The GLCM *P* (*i, j* | θ, *d*) calculates the number of times a pixel with gray -level ***i*** occurs with another pixel with a gray value ***j*** jointly. It is defined as the joint probability of specific pixels having certain gray -level values. The rotation angles of an offset are 0°, 45°, 90°, 135°, and the distance to the neighboring pixel is 1, 2, 3…; the same images have different co-occurrence distributions (21). The RLM *P r*(*i*,*j* |θ) represents the number of runs for pixels with gray level ***i*** and run length ***j*** for a given direction θ. The following ten features of RLM were derived: short run emphasis, long run emphasis, gray level non-uniformity, run length non-uniformity, low gray level run emphasis, high gray level run emphasis, short run low gray level emphasis, short run high gray level emphasis, long run low gray level emphasis, and long run high gray level emphasis (22, 23). The formulas for some parameters are displayed in Table 1. The total number of features extracted from this data is 385. Then, the AD or HC label was added for each subject.

**Table 1:** Definition of the features measures computed in this study after feature selection.

**Type of measure**	**Name**	**Formula**
Texture Parameter	ClusterShade_AllDirection_offset1	$\underset{i,j}{\sum }{\left(\left(i-\mu \right)+\left(j-\mu \right)\right)}^{3}g\left(i,j\right)$
GLCM Parameter	InverseDifferenceMoment_AllDirection_offset1	$f_{5}=\overset{N_{8}}{\underset{i=1}{\sum }}\overset{N_{8}}{\underset{j=1}{\sum }}\frac{1}{1+{\left(i-j\right)}^{2}}p\left(i,j\right)$
GLCM parameter	InverseDifferenceMoment_AllDirection_offset4_SD	$f_{5}=\overset{N_{8}}{\underset{i=1}{\sum }}\overset{N_{8}}{\underset{j=1}{\sum }}\frac{1}{1+{\left(i-j\right)}^{2}}p\left(i,j\right)$
RLM parameter	ShortRunEmphasis_angle45_offset1	$SRE\left(\theta \right)=\frac{1}{n_{r}}\overset{M}{\underset{i=1}{\sum }}\overset{N}{\underset{j=1}{\sum }}\frac{p\left(i,j,\theta \right)}{j^{2}}$
RLM parameter	RunLengthNonuniformity_AllDirection_offset4_SD	$RLN\left(\theta \right)=\frac{1}{n_{r}}\overset{N}{\underset{j=1}{\sum }}{\left(\overset{M}{\underset{i=1}{\sum }}p\left(i,j,\theta \right)\right)}^{2}$
RLM parameter	ShortRunHighGreyLevelEmphasis_AllDire ction_offset4_SD	$SRHGE\left(\theta \right)=\frac{1}{n_{r}}\overset{N}{\underset{j=i}{\sum }}\overset{M}{\underset{i=1}{\sum }}\frac{p\left(i,j,\theta \right)i^{2}}{j^{2}}$
RLM parameter	ShortRunEmphasis_angle90_offset7	$SRE\left(\theta \right)=\frac{1}{n_{r}}\overset{M}{\underset{i=1}{\sum }}\overset{N}{\underset{j=1}{\sum }}\frac{p\left(i,j,\theta \right)}{j^{2}}$
RLM parameter	LongRunEmphasis_AllDirection_offset4_SD	$LRE\left(\theta \right)=\frac{1}{n_{r}}\overset{M}{\underset{i=1}{\sum }}\overset{N}{\underset{j=1}{\sum }}p\left(i,j,\theta \right)j^{2}$
RLM parameter	ShortRunEmphasis_angle0_offset4	$SRE\left(\theta \right)=\frac{1}{n_{r}}\overset{M}{\underset{i=1}{\sum }}\overset{N}{\underset{j=1}{\sum }}\frac{p\left(i,j,\theta \right)}{j^{2}}$
RLM parameter	ShortRunEmphasis_angle90_offset4	$SRE\left(\theta \right)=\frac{1}{n_{r}}\overset{M}{\underset{i=1}{\sum }}\overset{N}{\underset{j=1}{\sum }}\frac{p\left(i,j,\theta \right)}{j^{2}}$
RLM parameter	GreyLevelNonuniformity_AllDirection_offset7_SD	$GLN\left(\theta \right)=\frac{1}{n_{r}}\overset{M}{\underset{i=1}{\sum }}{\left(\overset{N}{\underset{j=1}{\sum }}p\left(i,j,\theta \right)\right)}^{2}$

*For texture parameter, g is a GLCM, where i,j are the spatial coordinates of g (i,j). For GLCM parameters, i is a gray-level, j is a gray value, N is the number of classes of gray levels. For RLM parameters, n_r_ is the number of runs, N is the number of classes of gray levels, and M is the size in voxels of the largest region found*.

### Feature selection

The preprocessing before feature selection was divided into three steps. The first step was dealing with the abnormal value. Here, we replaced the abnormal values by mean. The second step was to set the data to the training data proportion of 0.7 and the testing data proportion of 0.3. The third step was to preprocess the training data after division and perform the same operation on the testing data. This method is known as standardization. The feature selection steps are as follows.

Step 1: The software first sought to identify the features that contribute to the result using the *T*-test (*P* < 0.05). The rank sum test was used to select the features with significant differences (*P* < 0.05), and the features of *T-*test and rank sum test were selected together.
Step 2: The correlation analysis reduced the dimension. The filter threshold was set to 0.9 for the Spearman rank correlation coefficient analysis that was conducted on any two feature columns. The two features were highly correlated if the correlation coefficient was > 0.9, thereby excluding of one of them.
Step 3: In the training data, the most useful features were selected by the least absolute shrinkage and selection operator (LASSO) Cox regression model. We need to minimize the sum of squares of residues, with the sum of the absolute values of the selected features coefficients being not more than a tuning parameter (λ). We chose the λ which got the minimum criteria according to 10-fold cross-validation in the LASSO model. This method was suitable for the regression analysis of high-dimensional data, and patient features could be selected based on the associations with the survival endpoints and time (24).

### Machine learning

Firstly, training data and testing data were loaded for the following up model building and testing. We subsequently selected the logical regression method to establish a classification model for AD diagnosis. This method was based on the linear function; it served as an independent variable into the sigmoid function. According to the probability *P* of the output (probability that the classification result is 1), the classification was determined. It's one of the machine learning methods.

## Results

### Comparison of demographic and neuropsychological performance

The demographic variables did not differ significantly between patients and control subjects, as assessed by SPSS (version 22.0). However, the neuropsychological performance was significantly different between the two groups (Table 2).

**Table 2:** Demographics performances of the AD and healthy controls.

	**AD group**	**HC group**	**Statistic**	***p* value**
Sample size	78	44	NA	NA
Age (years, mean ± SD)	69.18 ± 12.23	65.43 ± 9.70	−1.75	0.08
Gender (Male: Female)	25:53	20:24	2.17^*^	0.14^*^
Education (years, mean ± SD)	7.54 ± 4.16	7.09 ± 3.38	−0.61	0.54
MMSE	16.94 ± 5.94	29.14 ± 0.77	17.87	<0.01

SD standard deviation; Statistics were calculated with t tests unless otherwise indicated;

* *x^2^test was used; MMSE mini-mental state examination*.

### Feature selection results

**Figure 1:**
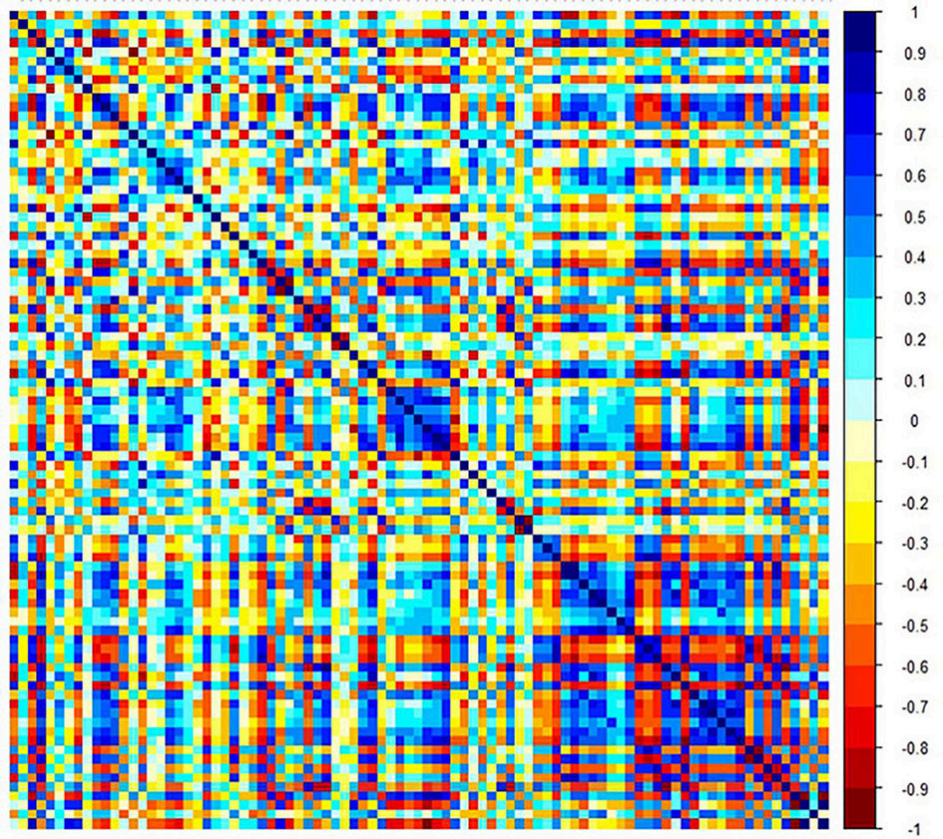
Graph shows correlation analysis between the parameters of training data.

**Figure 2:**
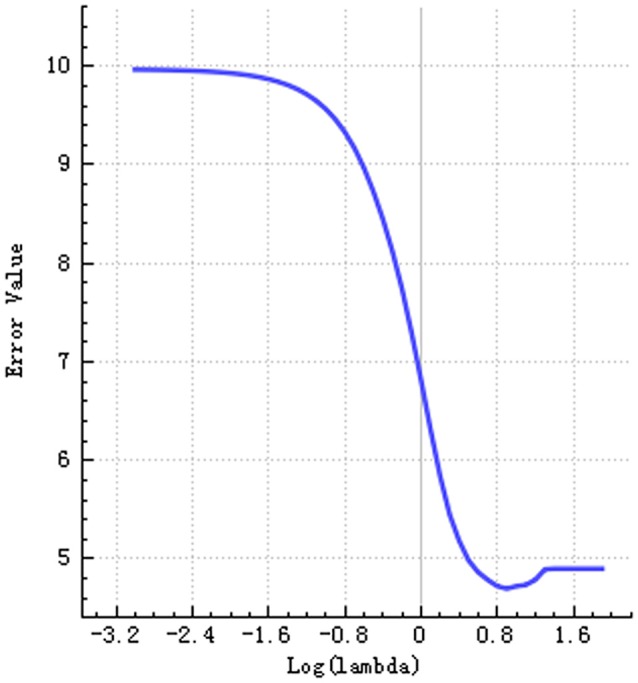
Graph shows error-lambda.

**Figure 3:**
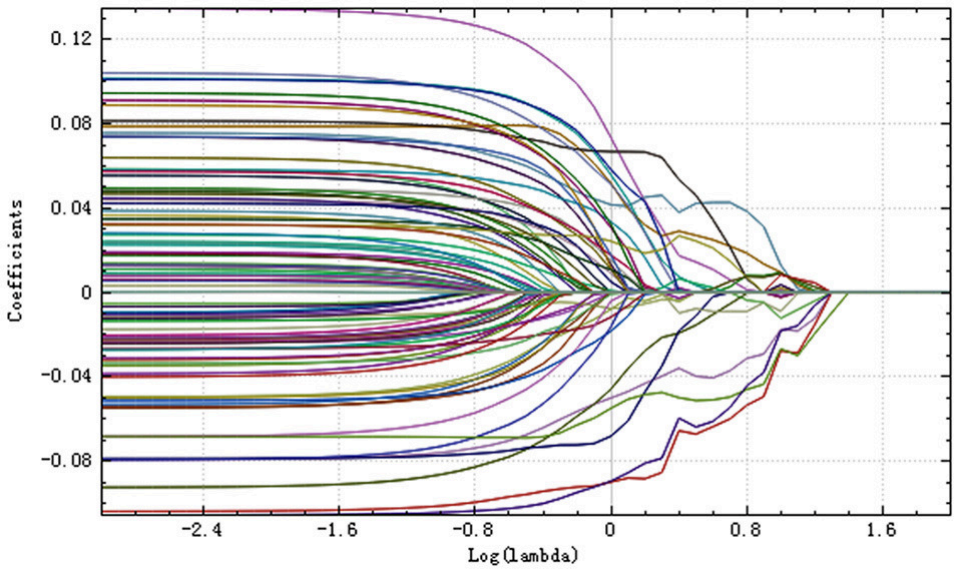
Plot of coefficients-lambda.

Step 1: A total of 385 features were extracted. The selective method was *T* test + MW. The remaining feature number was 196.
Step 2: The selective method was correlation analysis. The threshold value was 0.9, correlation method Spearman, and the remaining feature number was 89 (Figure 1).
Step 3: The selective method was Lasso. We found an optimal lambda by using cross-validation. The error-lambda graph is illustrated in Figure 2. The coefficients-lambda graph is shown in Figure 3. The remaining feature number was 11. The feature name order was as follows:
  “InverseDifferenceMoment_AllDirection_offset1”;
    “ClusterShade_AllDirection_offset1”;
    “ShortRunEmphasis_angle45_offset1”;
    “InverseDifferenceMoment_AllDirection_offset4_SD”;
    “RunLengthNonuniformity_AllDirection_offset4_SD”;
    “ShortRunHighGreyLevelEmphasis_AllDirection_offset 4_SD”;
    “ShortRunEmphasis_angle90_offset7”;
    “LongRunEmphasis_AllDirection_offset4_SD”;
    “ShortRunEmphasis_angle0_offset4”;
    “ShortRunEmphasis_angle90_offset4”;
    “GreyLevelNonuniformity_AllDirection_offset7_SD” (Table 1).

### Machine learning results

**Figure 4:**
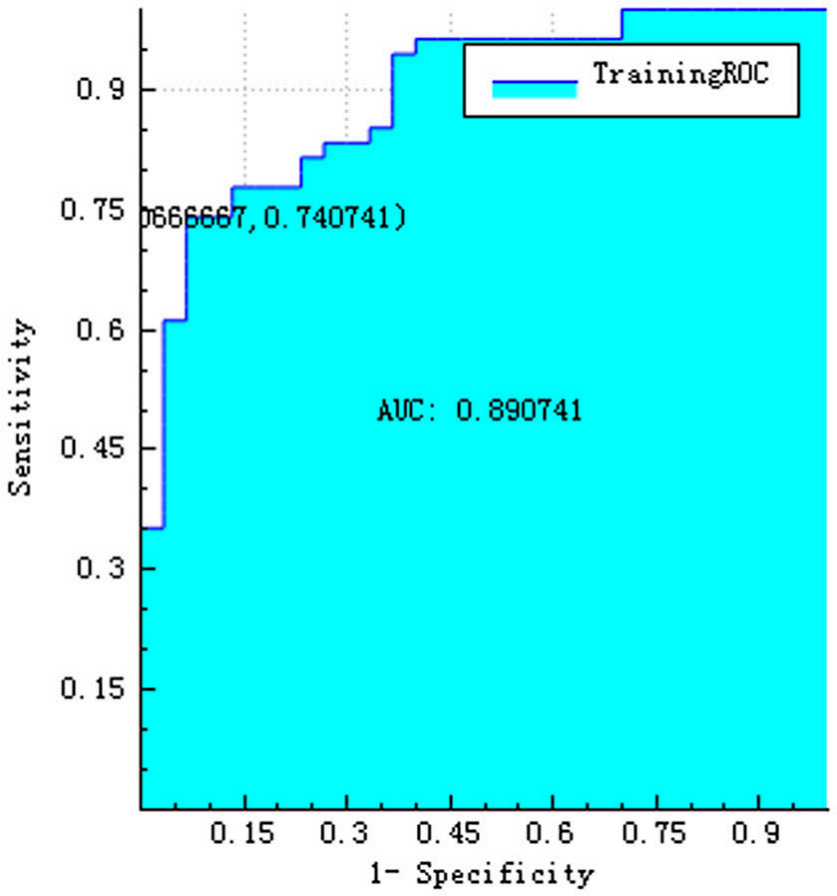
ROC curve of training data.

**Figure 5:**
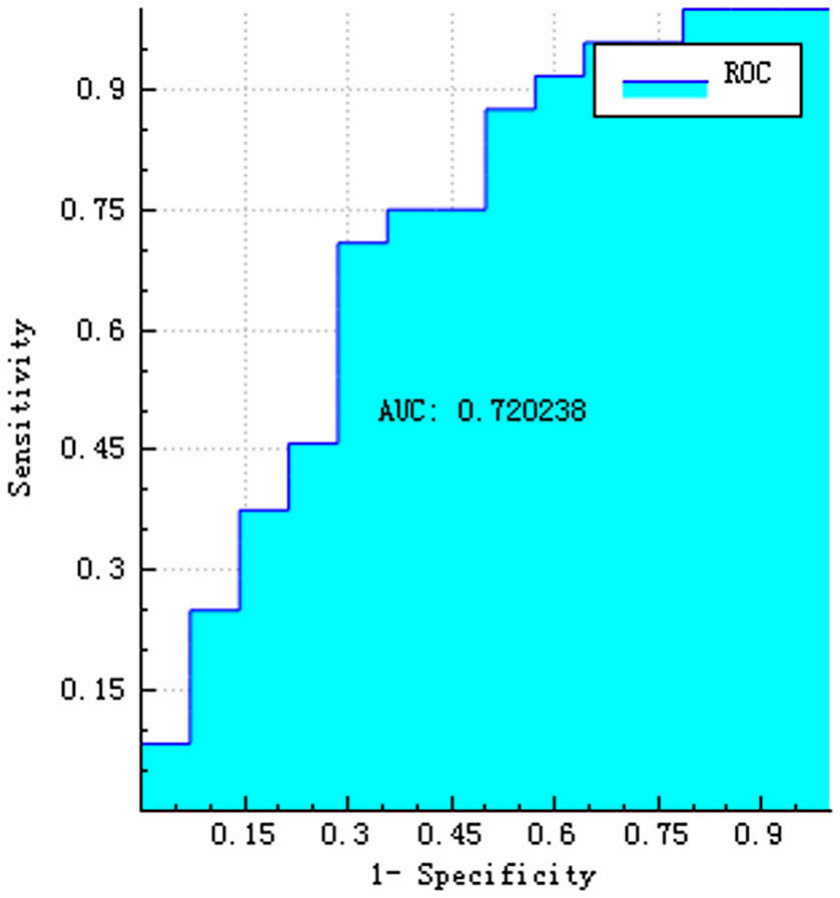
ROC curve of testing data.

**Figure 6:**
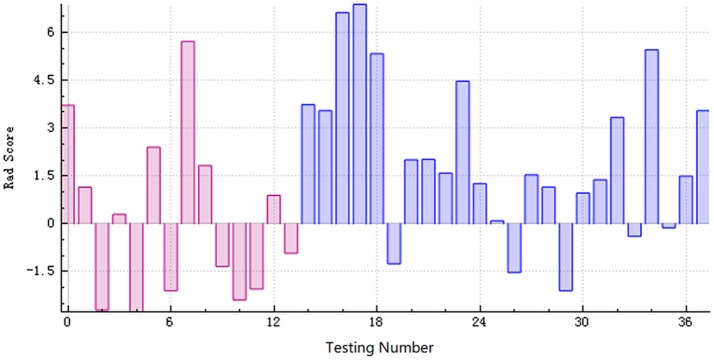
The radiomics score based on the testing data. The red area below the horizontal line and the blue area above the horizontal line represented the accurate prediction. On the contrary, the red area above the horizontal line and the blue area below the horizontal line represented the false prediction.

The training and testing data were loaded, the proportion of the training data was 0.7, while that of the testing data was 0.3. While establishing the classifier discriminating the patients with AD from HC subjects, the selected method was logistic regression based on the selected features. The area under the receiver operating characteristic curve values (AUC), sensitivity, specificity, accuracy, precision, positive predictive value, and negative predictive value were 0.720, 0.792, 0.500, 0.684, 0.731, 0.731, and 0.583, respectively (Figures 4–6).

## Discussion

The major finding of the present study was that the CC radiomics-based classification model discriminated the patients with AD from HC subjects. After a three-step feature selection, an 11-feature radiomics signature was constructed using logistic regression model for the diagnosis of patients with AD. Although the specificity of this model was not extremely high, its diagnostic value was better than the other indicators.

The radiomics analysis has already been applied to neuropsychiatric disorders. For example, a radiomics study found texture differences between autism spectrum disorder and control groups in the right hippocampus, left choroid-plexus, CC, and cerebellar white matter (25). Another recent radiomics study indicated that cerebral morphometric alterations can allow discrimination between the patients with attention deficit hyperactivity disorder and control subjects and also among the subtypes (10). This study has built random forest classifiers for diagnosis and subtyping. In addition, textures differences in the CC and thalamus were observed in AD and amnestic mild cognitive impairment (3). One study investigated the three-dimensional texture as a putative diagnostic marker of AD (26). However, currently, there is no study describing the establishment of the model in the analysis of AD radiomics studies. Thus, for the first time, the present study attempted to construct a classification model for the diagnosis of AD. In addition, the machine learning method was added to the modeling.

The CC presented abnormality in the surface-based morphometry and microstructural integrity in the patients with AD (27). Another study found significant volume reductions in anterior and posterior of the CC in severe AD patients (28). A voxel-based morphometry study in AD detected significant atrophy of CC in the anterosuperior splenium, the anterior and posterior portions of the body, and the rostral portion of the genu (29). The volume changes in the different portions of the CC might exist in different pathological processes. Reportedly, the anterior portion of CC consists of myelinated axons with a small diameter; however, the posterior portion consists of thick fibers (1). Thus, this abnormal development might result in the differences observed in the texture. In the current study, the texture features derived from the CC were used for differentiating between AD and HC subjects. Herein, we established an analysis framework on the basis of CC radiomics and machine learning methods for AD diagnosis, which suggested that the CC radiomics features could be used as biomarkers for AD diagnosis. Nevertheless, longitudinal developmental studies are essential to substantiate these interpretations. Structural data were involved in the process of classifier building, thereby providing a neuroanatomical evaluation of the disorder.

The radiomics signature consisted of 11 imaging features that were deep features, extracted from the three-dimensional T1-weighted MPRAGE images. The deep features extracted from A.K. performed better than the conventional handcrafted features in the diagnosis of patients with AD. As expected, the deep features reflected higher order imaging patterns and captured more imaging heterogeneity as compared to the low-level shape, intensity, and texture features. Cluster Shade is one of the texture parameters. Cluster analysis is the task of grouping objects such that the objects in the same cluster are rather similar to each other than those in the other clusters. The inverse difference moment is one of the GLCM parameters. Short run emphasis, long run emphasis, run length non-uniformity, short-run high gray level emphasis, and gray level non-uniformity constitute the RLM parameters. They reflect the measurement of nonuniformity of the length and that of the grayscale. Thus, the observed abnormalities in the CC may be clinically relevant with respect to cognitive and behavioral issues in patients with AD. However, the relationship between the radiomics features and the genetic characteristics is yet challenging.

Nevertheless, the present study has several limitations. First, owing to the insufficient sample size, the classification performance may be limited. Thus, a large-scale multicenter study is required to fully assess the generalization ability of the radiomics model in future. Second, although no statistically significant difference was detected between the two groups in the sex ratio analysis, we did not achieve a complete 1:1 match, and hence, it was not possible to completely exclude the effect on the study results. Finally, there is no evaluation of white matter integrity using white matter imaging method, such as DTI and DKI, which need to be further studied.

Future radiomics work can use additional imaging modalities, such as diffusion tensor imaging and functional MRI. These radiomics models might contain additional anatomical structures related to AD, such as the hippocampus, medial temporal lobe, thalamus, as well as, the whole brain. Furthermore, we can improve the classification performance by combining the radiomics analysis with established clinical risk factors such as age and MMSE score.

In conclusion, our findings indicated that a moderately successful diagnostic classification efficiency could be achieved between patients with AD and HC subjects using the CC radiomic features. The workflow was automatic, and therefore, potentially useful in the clinical setting. As a non-invasive MR-based imaging biomarker, the radiomics analysis might provide a valuable and practical method to identify the patients with AD and guide the individualized treatment.

## Ethics statement

We confirm that we have read the Journal's position on issues involved in ethical publication and affirm that this report is consistent with those guidelines.

## Author contributions

QF, HJ, DM, EY, and ZD designed the study. ZL and QF collected patient data and provided clinical expertise. QF and MW segmented the MR images. QF drafted the manuscript. YC interpreted the data for the work. All the authors discussed the results and read and approved the final version of the manuscript.

### Conflict of interest statement

The authors declare that the research was conducted in the absence of any commercial or financial relationships that could be construed as a potential conflict of interest.
